# Evaluation of the positional reproducibility of sedation versus non-sedation state in pediatric radiotherapy: a retrospective study

**DOI:** 10.3389/fonc.2024.1475060

**Published:** 2024-10-29

**Authors:** Zhanquan Lei, Yuequan Shi, Yiqun Liu, Bo Gao, Kongfeng Shao, Xijin Lin, Lizhen Wu, Zhaojie Yao

**Affiliations:** ^1^ Radiotherapy, Fujian Children’s Hospital, Fuzhou, China; ^2^ Radiology, Fujian Maternity and Child Health Hospital, Fuzhou, China

**Keywords:** radiotherapy, sedation, cone-beam computed tomography, tumor, positional deviations

## Abstract

**Objective:**

To assess the positional reproducibility of sedated and non-sedated pediatric tumor patients during radiotherapy through a retrospective analysis of cone-beam computed tomography (CBCT) and planned computed tomography (CT) scan data.

**Methods:**

The positional reproducibility of 40 pediatric tumor patients, aged 2 to 17 years with a median age of 4.5 years, who received radiotherapy under sedated and non-sedated states was retrospectively compared. The first CBCT images obtained during CT-based treatment planning were analyzed. The analysis encompassed six-dimensional positional changes, including vertical (Vrt), longitudinal (Lng), lateral (Lat), rotational (Rtn), pitch, and roll directions. Kolmogorov-Smirnov Z nonparametric rank-sum testing was employed to evaluate the positional deviations, considering absolute values regardless of directionality. Data were further stratified based on different fixation methods used during treatment.

**Results:**

Sedated patients exhibited significantly smaller positional deviations in Vrt, Lng, Lat, and Rtn directions in the body membrane group compared with their non-sedated counterparts (P<0.05). Similarly, sedated patients demonstrated reduced positional deviations in Vrt, Lng, Lat, Rtn, pitch and Roll directions in the head and neck group compared with non-sedated patients (P<0.05). Meanwhile, compared with vacuum bag plus body membrane fixation, the head and shoulder film fixation technique proved superior in terms of positional reproducibility during sedated treatment, specifically in Vrt, Lng, Lat, Pitch, and Roll directions (P<0.05). Similarly, compared with the alternative fixation method, the head and shoulder film fixation method showed better positional deviations in six-Dimensional directions in non-sedated patients (P<0.05).

**Conclusion:**

While sedated radiotherapy may offer advantages in terms of positional reproducibility, the present study underscores the importance of considering non-sedated radiotherapy as a viable option for pediatric tumor patients. Non-sedated treatment not only provides effective tumor control but also mitigates the psychological trauma and long-term side effects associated with repeated sedative drug use. Future studies should further explore the optimal sedation and fixation strategies for pediatric radiotherapy.

## 1 Introduction

Malignant tumors are among the leading diseases that threaten human health globally, with recent years witnessing a significant rise in the incidence of pediatric malignancies. Given the sensitivity of most pediatric tumors to X-rays, radiotherapy has become a crucial component in the comprehensive treatment of pediatric tumors (1). Recent advances in radiotherapy techniques and equipment have increased treatment prospects for malignant tumors. The emergence of advanced techniques such as Intensity-Modulated Radiation Therapy (IMRT) and Volumetric Modulated Arc Therapy (VMAT) has further enhanced the precision and safety of radiotherapy (2).

However, the use of high-energy radiation during radiotherapy requires pediatric patients to undergo treatment alone in enclosed accelerator rooms. This treatment environment often leads to decreased radiotherapy positioning accuracy due to psychological factors such as fear and loneliness among pediatric patients. This positioning inaccuracy can result in deviations in radiation dose distribution from the predetermined treatment plan, affecting treatment outcomes, increasing the risk of complications, and potentially leading to treatment interruption (3, 4).

To ensure the precision of radiotherapy, sedatives such as chloral hydrate, dexmedetomidine, and phenobarbital are commonly used clinically to treat patients in a sedated state. However, repeated use of sedatives can lead to long-term complications, such as cognitive impairment and behavioral changes, due to drug accumulation. Although sedatives have significantly improved pediatric tumor outcomes, these benefits have been counteracted by long-term side effects and their impact on the pediatrics patient’s quality of life (5).

Consequently, numerous radiotherapy centers have begun exploring non-pharmacological approaches such as psychological intervention, simulation training, playing soothing music, play and video-based distraction (6, 7). to promote the completion of radiotherapy in non-sedated pediatric patients. It remains uncertain whether radiotherapy positioning can be consistently accurate and reliable in a non-sedated patient, even with the use of advanced techniques. This question has become a focus of recent research (8, 9).

This study aimed to assess the accuracy and consistency of radiotherapy positioning in children who were not sedated during treatment. By examining cone-beam computed tomography (CBCT) and planned computed tomography (CT) scan data, the researchers sought to provide guidance for various treatment options in radiation therapy for pediatric cancer patients. This treatment approach can reduce patient discomfort, minimize the risk of complications, and enhance treatment outcomes and the quality of life of patients.

## 2 Patients and methods

### 2.1 Ethics approval

TGiven that this study was based on existing data and did not involve direct patient interaction, the requirement for informed consent was waived. Therefore, it was approved by the Institutional Review Board of Fujian Children’s Hospital, ensuring ethical compliance. (Approval Number: [2024ETKLRK070002]).

### 2.2 Patients

Children who received radiation therapy between August 2022 and April 2024 were included in this retrospective study. Patients were excluded if they had: (1) To mitigate the risk of inaccuracies arising from technical discrepancies among therapists in the utilization of special positioning fixation devices, hereby necessitating their exclusion from the study(e.g. individuals undergoing stereotactic radiosurgery (SRS) who necessitated the utilization of specialized head mounts, positioning films, and head rests to ensure precise delivery of radiation); (2) a Karnofsky performance status score below 80 before treatment; (3) unclear diagnosis, (4) In the context of radiotherapy administration, patients with claustrophobia may experience panic attacks or severe psychophysiological reactions, encompassing accelerated heart rate and rapid breathing, thereby necessitating their exclusion from the study; (5) did not complete their radiation therapy. Ultimately, 40 patients (21 boys and 19 girls, with an average age of 4.5 years) were included in the final analysis.

### 2.3 Methodology

Simulation and positioning were performed using a 16-slice wide-bore CT simulator (GE Discovery RT590,US),while patient immobilization was achieved utilizing an integrated immobilization system (Huayuxin HYX-UTS-CM,Ji’nan, CN).Treatment delivery and CBCT image scanning were performed using a Varian TrueBeam medical linear accelerator (Varian Medical Systems, Palo Alto, CA). The Eclipse Treatment Planning System (Eclipse TPS, version 15.5, Varian Medical Systems, Palo Alto, CA) was used to plan radiotherapy treatments for patients.

For patients with treatment sites in the torso, a 1-meter-long vacuum bag combined with a body thermoplastic mask was used for immobilization. Patients were positioned in a supine position in the vacuum bag with their hands raised and breathed normally. For head and neck immobilization, we employed an S-shaped head and shoulder support system incorporating a head and shoulder mask illustrated. CT simulation was performed to obtain planning CT images with a slice thickness of 2.5 mm. Accurate patient reference coordinates (x, y, z) were marked using 1 mm lead markers, and the planning CT images were sent to the TPS.

All radiotherapy plans were subjected to the following steps: (1) target delineation and delineation of organs at risk (OARs) by radiation oncologists, which were reviewed by senior or attending physicians; (2) selection of appropriate radiotherapy techniques by physicists based on the prescribed requirements; (3) review and confirmation of the plans by senior doctors and physicists to ensure compliance with radiotherapy standards; (4) verification of the plans by physicists to achieve a gamma pass rate (3%, 3 mm) of ≥95% before starting radiotherapy.

The 40 enrolled patients were classified based on different positioning techniques and the use of sedatives during treatment as follows: a) head and neck group (head and neck mask or U-shaped face mask fixation): sedated/non-sedated; b) body membrane group (vacuum bag and body membrane fixation): sedated/non-sedated (patients receiving total central nervous system irradiation as part of total central nervous system radiation therapy were included in the head and neck group, while those receiving spinal irradiation were included in the body membrane group). For children younger than 8, psychological interventions, music, videos, and playing games were used in the treatment room before simulation positioning to reduce their fear of radiation therapy. Children who could maintain the radiotherapy position for 10-15 minutes using these methods received non-sedated radiotherapy.

**Figure 1:**
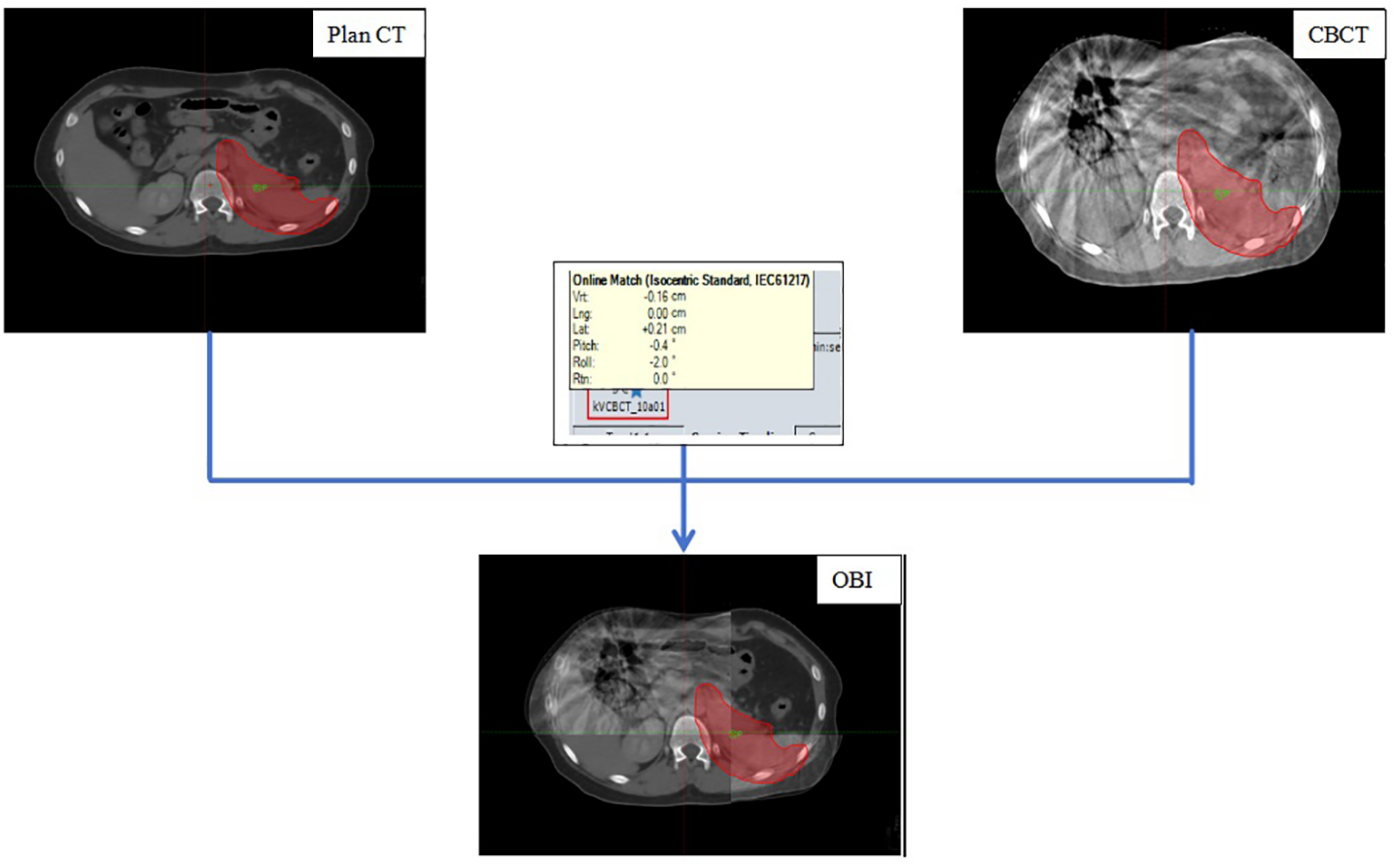
Pretreatment On-Board Imaging (OBI) Registration Process.

All patients underwent CBCT scanning before the first five radiotherapy sessions. Online On-Board Imaging (OBI) matching technology was used to align the region of interest, defined by the outer body contour and bony landmarks, with the planning CT images. This process, illustrated in **Figure 1**. After the registration results met clinical treatment requirements, six-dimensional deviation data, including superior-inferior (vertical [Vrt]), left-right (longitudinal [Lng]), anterior-posterior (lateral [Lat]), rotation around the Z axis (lateral [Rtn]), rotation around the X-axis (Pitch), and rotation around the Y-axis (Roll), were recorded. The six-dimensional treatment bed was then moved to the correct position. Finally, the registration data were saved and treatment was started.

### 2.4 Statistical analysis

Data analysis was performed using SPSS 24.0 software. Categorical variables were analyzed using the chi-square test. Normally distributed variables were expressed as the median [interquartile range] (M [Q1, Q3]). Kolmogorov-Smirnov Z nonparametric rank-sum testing was employed to evaluate the positional deviations, considering absolute values regardless of directionality. P<0.05 was considered statistically significant.

## Results

All 40 pediatric patients completed the course of radiation therapy, of whom 20 underwent radiation therapy after sedation; 6 patients were treated with intensity-modulated radiation therapy (IMRT) and 34 with volumetric modulated arc therapy (VMAT). The baseline characteristics of patients are presented in **Table 1**.

**Table 1:** Patient baseline characteristics.

	Sedation	Non-sedation
Gender		
Male	11	10
Female	9	10
Age (y)		
Mean age	3.4	8.1
Median age	3.0	7.0
Fixation method		
Head and shoulder mask	10	10
Body mask and vacuum bag	10	10
Type of tumor		
Meningioma	2	0
Glioma	2	1
Ewing’s Sarcoma	2	2
Wilm’s tumor	0	1
Pinealoblastoma	3	0
Hodgkin’s lymphoma	0	1
Neuroblastoma	5	0
Germinoma	1	2
Rhabdomyosarcoma	1	2
Myoblastoma	3	5
Teratoma	1	6
Irradiation technique		
IMRT	2	4
VMAT	18	16

**Figure 2:**
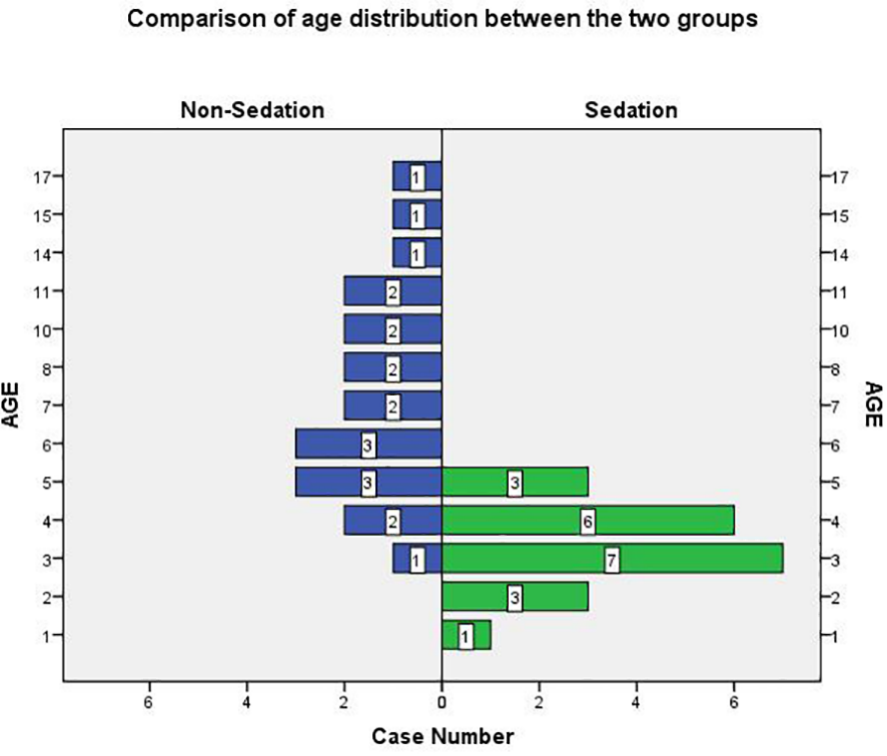
Comparison of age distribution between the two groups.

In both groups of pediatric patients, 29 were aged 3 to 8 years, and 13 of these (44.8%) received non-sedated radiotherapy. The age distribution of the patients in both groups is shown in **Figure 2**.

**Figure 3:**
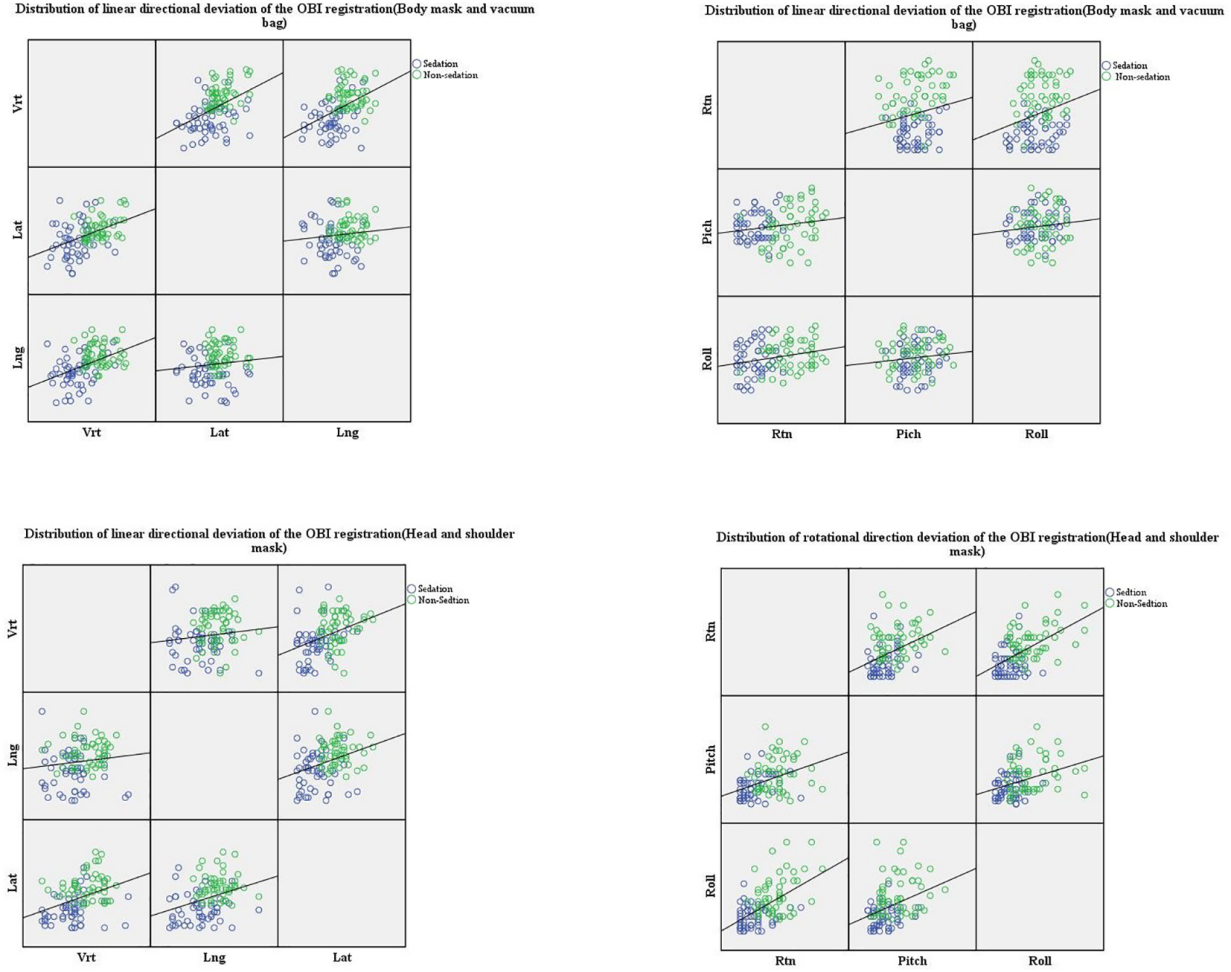
Six-Dimensional Deviation Distribution Analysis Between Two Patient Groups.

The distributions of positional deviations in the six-dimensional directions during OBI registration for the two patient groups are shown in **Figure 3**. In the sedation group, the maximum position deviation in the linear direction was 0.34 mm, and the maximum angular deviation in the rotational direction was 3°. In the non-sedation group, the maximum position deviation in the linear direction was 0.44 mm, and the maximum angular deviation in the rotational direction was 2.5°.

In the body mask group, Patients who underwent sedation radiotherapy exhibited smaller positioning deviations in Vrt, Lng, Lat, and Rtn directions compared with those who received non-sedation radiotherapy. The differences were statistically significant (P<0.05). However, no statistically significant differences were observed in the Pitch and Roll directions between the two groups (P>0.05) (**Table 2**).

**Table 2:** Comparison of the difference in positioning correction between sedative and non-sedative positioning in the body membrane group.

	Vrt (cm)	Lng (cm)	Lat (cm)	Rtn (°)	Pitch (°)	Roll (°)
Sedation	0.157 (0.108, 0.210)	0.167 (0.110, 0.210)	0.193 (0.118, 0.263)	0.476 (0.100, 0.700)	0.838 (0.500, 1.100)	1.216 (0.900, 1.500)
Non-sedation	0.296 (0.238, 0.332)	0.267 (0.218, 0.330)	0.264 (0.218, 0.303)	1.546 (1.100, 2.100)	1.428 (1.075, 1.800)	1.404 (1.100, 1.700)
Z	3.500	2.800	2.500	3.800	0.900	1.000
P	0.000	0.000	0.000	0.000	0.393	0.270

In the Head and Shoulder mask group, patients who received sedation radiotherapy had smaller positional deviations in all six-dimensional directions compared to those who received non-sedated radiotherapy. These differences were statistically significant (P<0.05) (**Table 3**).

**Table 3:** Comparison of the difference in positioning correction between sedative and non-sedative positioning in the head and neck group.

	Vrt (cm)	Lng (cm)	Lat (cm)	Rtn (°)	Pitch (°)	Roll (°)
Sedation	0.110 (0.070, 0.130)	0.100 (0.038, 0.143)	0.070 (0.038, 0.123)	0.250 (0.100, 0.500)	0.500 (0.200, 0.725)	0.450 (0.175, 0.700)
Non-sedation	0.155 (0.110, 0.200)	0.160 (0.120, 0.190)	0.180 (0.148, 0.213)	0.950 (0.700, 1.225)	1.000 (0.500, 1.500)	1.100 (0.700, 1.700)
Z	2.300	2.300	3.300	3.100	1.900	2.700
P	0.000	0.000	0.000	0.000	0.001	0.000

During the sedation treatment, the positioning deviation in Vrt, Lng, Lat, pitch and Roll directions was lower for the head and shoulder membrane fixation method than for the vacuum bag plus body membrane fixation method. Differences were statistically significant (P<0.05) (**Table 4**).

**Table 4:** Comparison of the difference in positioning deviation during sedative radiotherapy between different body position fixation methods.

Fixation method	Vrt (cm)	Lng (cm)	Lat (cm)	Rtn (°)	Pitch (°)	Roll (°)
Head and shoulder mask	0.110 (0.070, 0.130)	0.100 (0.038, 0.143)	0.070 (0.038, 0.123)	0.250 (0.100, 0.500)	0.500 (0.200, 0.725)	0.450 (0.175, 0.700)
Body mask and vacuum bag	0.157 (0.108, 0.210)	0.167 (0.110, 0.210)	0.193 (0.118, 0.263)	0.476 (0.100, 0.700)	0.838 (0.500, 1.100)	1.216 (0.900, 1.500)
Z	1.800	2.200	2.600	1.100	4.00	3.500
P	0.003	0.000	0.000	0.178	0.000	0.000

During the non-sedation treatment, the positioning deviation in All six-dimensional directions was significantly lower for the head and shoulder membrane fixation method than for the vacuum bag plus body membrane fixation method. Differences were statistically significant (P<0.05) (**Table 5**).

**Table 5:** Comparison of the difference in positioning deviation during non-sedative radiotherapy between different body position fixation methods.

Fixation method	Vrt (cm)	Lng (cm)	Lat (cm)	Rtn (°)	Pitch (°)	Roll (°)
Head and shoulder mask	0.155 (0.110, 0.200)	0.160 (0.120, 0.190)	0.180 (0.148, 0.213)	0.950 (0.700, 1.225)	1.000 (0.500, 1.500)	1.100 (0.700, 1.700)
Body mask and vacuum bag	0.296 (0.238, 0.332)	0.267 (0.218, 0.330)	0.264 (0.218, 0.303)	1.546 (1.100, 2.100)	1.428 (1.075, 1.800)	1.404 (1.100, 1.700)
Z	4.100	3.500	2.800	2.300	1.800	1.500
P	0.000	0.000	0.000	0.000	0.003	0.022

## Discussion

This study examined the consistency of patient positioning in 40 pediatric patients aged 18 and younger. The patients were retrospectively selected and analyzed based on their use of sedatives and different body immobilization techniques before radiation therapy. The results showed that the registration deviations of the CBCT scan, conducted prior to the first five radiotherapy sessions, were <5 mm in the linear direction for the body film group and <3 mm for the head and neck group. Both groups exhibited <3° rotations in the rotational direction. meeting the standards established by authoritative international agencies such as the China National Cancer Center, the American Association of Physicists in Medicine (AAPM), and the Radiation Therapy Oncology Group (RTOG).

IMRT has become a primary technique in tumor radiotherapy due to its ability to deliver high doses to the target while minimizing radiation exposure to surrounding OARs (10, 11). However, IMRT involves the use of multiple small subfields, and any positioning errors before treatment can affect the target dose distribution, potentially increasing side effects or reducing tumor control (12). Accurate positioning for each radiotherapy treatment is crucial for precise radiation delivery (13, 14). Studies by Anees Dhabaan et al (15) and Hattel et al (16) using surface-guided radiation therapy (SGRT) systems observed that patients experienced deviations in both translational and rotational directions during real-time monitoring. According to Zumsteg and colleagues, using the first five CBCT shifts to adjust subsequent radiation therapy fractions reduced the number of fractions requiring correction to 19% of all delivered fractions. Additionally, the percentage of patients with average daily 3D errors exceeding 5 mm decreased from 35.7% to 14.3% compared to no image guidance. However, using an average of the first 10 CBCT shifts did not significantly improve these results (17). The results of this study exhibit a substantial concordance with preceding research endeavors, underscoring the pivotal significance of image-guided radiation therapy (IGRT) in refining treatment efficacy and mitigating the specter of complications, particularly in the realm of pediatric patient management, intricate anatomical territories, and tumors exhibiting substantial mobility. As medical imaging technologies forge ahead at an unprecedented pace, the integration of advanced modalities, such as PET-CT and MRI, into IGRT protocols furnishes multifaceted insights for precise tumor targeting and individualized treatment strategies. Consequently, it is paramount to scrutinize the long-term ramifications of IGRT practices on patient prognosis and quality of life. By leveraging retrospective analyses and prospective clinical trials, we can meticulously evaluate the impact of IGRT on bolstering local control, diminishing recurrence rates, and enhancing patient survival outcomes, thereby reinforcing clinical decision-making and nurturing a more optimistic treatment landscape imbued with hope for patients.

Our results showed that, although there were some differences in positioning deviations in different directions after sedation and non-sedation radiotherapy, the deviations were still within the range that could be compensated by the six-dimensional treatment bed to achieve precise radiotherapy. During sedated treatment, positioning deviations in Vrt, Lng, Lat, and Rtn directions were lower for patients who underwent sedation radiation therapy than for those who did not. However, all these deviations were still within the acceptable range for precise radiotherapy. Additionally, different body fixation methods also resulted in different positioning deviations. During sedated treatment, the positioning deviations in Vrt, Lng, Lat,Pitch and Roll directions were lower for patients who were fixed with a head, neck, and shoulder film than for those fixed with a vacuum bag and body film but with no statistical significance in other directions was found between the two approaches. During non-sedated treatment, positioning deviations in six-dimensional directions were significantly lower for patients who were fixed with a head, neck, and shoulder film than for those fixed with a vacuum bag and body film, and the difference was statistically significant. Controlling the patient body position under the interference of external factors such as body fixation and laser lights proved difficult under non-sedated conditions due to the young age and clear consciousness of the patients, leading to slight body movements, which were more pronounced in the body film group. Conversely, under sedated conditions, patients were in a semi-conscious state with weaker active consciousness, resulting in relatively smaller positioning deviations. Choosing the right immobilization devices during patient positioning simulation is essential. Jared Becksfort at al (18) found that pediatric patients with head and neck radiation can experience reduced positional deviations when using a head-neck-shoulder immobilization mask. However, for radiation fields below the clavicle, the limitations of the head-neck-shoulder mask require the use of vacuum bags and body molds for immobilization. This approach helps to minimize positional deviations during radiotherapy, improving the accuracy and effectiveness of the treatment. Notably, keeping the patient in a more comfortable position during the body film production stage is essential to reduce deviations caused by positioning during treatment.

Many radiotherapy centers are currently focusing on research to reduce the use of sedative drugs in pediatric cancer patients, particularly young children, while maintaining or improving the accuracy and consistency of their positioning during treatment (19–21). McCoola B et al (22) reported that additional play appointments for pediatric patients aged 3-8 years can reduce the need for general anesthesia (GA) during treatment. Hess CB et al (23) investigated a positioning method for precise radiotherapy in pediatric tumor patients, finding that psychological induction can encourage active patient cooperation and accurate positioning. Joosse IR et al (24) addressed the challenges of maintaining the treatment position for a long time in pediatric tumor radiotherapy. They achieved positive outcomes by providing timely psychological support and guidance to children and their families. Wang et al (25). proposed that use of the “kindergarten effect” can improve the quality of life and stabilize the psychological activities of children while providing precise radiotherapy, improving patient compliance and treatment efficiency. Encouragingly, the youngest patient in the non-sedated radiotherapy group in the present study was 3 years. Therefore, younger pediatric cancer patients undergoing radiation therapy can achieve non-sedated radiation therapy through patient technician guidance, psychological intervention, simulation training, playing soothing music, play and video-based distraction and active cooperation from family members.

Nonetheless, this study has some limitations, such as a small sample size and a short follow-up period.Given these findings, further large-scale studies with long-term follow-up are necessary to fully understand the recurrence rates of tumors in different patient groups and the potential long-term side effects of sedation in children, including mental retardation, psychiatric problems, and motor coordination issues. This will provide a more complete picture of the treatment outcomes and associated risks.Our study excluded a subset of patients who underwent radiotherapy under sedation, based on predefined exclusion criteria. This exclusion criterion, while necessary to maintain the homogeneity of our sample and the focus of our analysis, potentially introduced a selection bias that may limit the generalizability of our findings. To validate our observations and broaden the applicability of our results, future research endeavors should endeavor to incorporate larger and more diverse cohorts, encompassing both patients treated with and without sedation during radiotherapy, thereby mitigating the potential impact of selection bias on the interpretation of study outcomes.

## Conclusions

Sedatives are commonly used in pediatric oncology patients to ensure accurate radiotherapy. However, prolonged sedative use can increase clinical risks for children. This retrospective study demonstrated that a subset of pediatric patients aged 3-8 could successfully receive precise radiotherapy without sedation by implementing pre-treatment interventions and the support of CBCT image-guided technology. This approach offers a more humane experience for patients and their families. Furthermore, the study revealed variations in positional deviations associated with different fixation devices, emphasizing the importance of selecting the appropriate device during simulation positioning.

## Data Availability

Publicly available datasets were analyzed in this study. This data can be found here: The datasets used and materials during the current study are available from the corresponding author on reasonable request. Email: 455676689@QQ.com.
